# Editorial: Modulation of Reward Circuitry by Pain and Stress

**DOI:** 10.3389/fpsyt.2017.00296

**Published:** 2017-12-22

**Authors:** Anna M. W. Taylor, Catherine M. Cahill

**Affiliations:** ^1^Department of Pharmacology, University of Alberta, Edmonton, AB, Canada; ^2^Department of Psychiatry and Biobehavioral Science, University of California Los Angeles, Los Angeles, CA, United States

**Keywords:** chronic pain, affect, stress, physiological, motivation and emotion, reward processing

**Editorial on the Research Topic**

**Modulation of Reward Circuitry by Pain and Stress**

Acute pain is a necessary, physiologically relevant phenomenon that serves as a warning signal for actual or potential tissue damage. It engages a complex and dedicated circuitry within the peripheral and central nervous system to signal and control the sensation of pain. Importantly, it is generally well managed by established analgesics, such as NSAIDs or opioids. Chronic pain, on the other hand, persists beyond any physiologically relevant purpose and becomes a disease state in and of itself. We now understand that chronic pain leads to adaptations in the sensory nervous system that distinguishes it from acute pain—a phenomenon that may contribute to its stubborn persistence in the face of the typical pharmacological armory.

One limitation to the classical approach to treatment of pain is the myopic focus on the sensory aspects of pain. However, pain has an equally important affective component. Over long periods of pain stimuli, engagement of affective or reward circuitry can impact underlying mood states, as illustrated by the fact that depression is one of the most common co-morbidities with chronic pain. Moreover, stress, which commonly accompanies on-going pain, can also modulate reward circuitry and has been implicated in prescription analgesic addiction and drug relapse. An emerging question is whether chronic pain can also induce adaptations in reward circuits that contribute to the severity and chronicity of this challenging disease, and the impact this may have on opioid misuse disorders.

This Research Topic includes several original research and review articles discussing the impact of chronic pain on reward circuitry. They provide evidence for how chronic pain changes reward circuitry, its interactions with the stress system, and the implications this has for the genesis of psychiatric disorders such as depression and addiction.

The review by Dos Santos et al. provides a thorough introduction to the neuroanatomical underpinnings of pain and reward. Evidence is presented from both clinical and preclinical chronic pain studies suggesting significant neuroadaptations within these circuits that may contribute to the chronification of pain and mental co-morbidities such as depression and anxiety. Strategies that restore normal function of these circuits may be beneficial in treating both the sensory and emotional symptoms associated with chronic pain.

This review is complemented nicely by the original research article by Wang et al. In this study, the authors explored changes in cortical activation following acute or chronic pain. Using DeltaFosB as a marker of neuronal activation, they found chronic, but not acute pain, stimulated DeltaFosB expression in the medial prefrontal cortex. This provides further evidence for activity-related changes in circuits involved in reward and motivation in chronic pain. The DeltaFosB activation in the medial prefrontal cortex was also observed in a chronic stress model (maternal separation), highlighting the shared pathologies between chronic pain and stress paradigms.

Engagement of reward circuits in chronic pain will undoubtedly impact a myriad of pain-related behaviors, including pain hypersensitivity and negative affect. In the review by Gandhi et al., the authors argue eloquently that disparate activity of reward circuitry contributes to the various coping strategies employed by those in pain. Beginning with a review of the fundamental principles and underlying neurobiology of active and passive pain coping strategies, the authors compare these processes between acute and chronic pain scenarios. Evidence is presented that suggests adaptations in reward circuitry of chronic pain patients interferes with the decision-making process and impedes active coping strategies.

Finally, the last two review articles tackle the issue of chronic pain and opioid misuse. In the review by Ghitza, the author takes a clinical perspective while reviewing the shared pathological adaptations within reward circuitry following repeated pain stimuli or opioid use. For example, chronic pain evokes increases in activity within the extended amygdala that may heighten the sensitivity to psychological stressors—a phenomenon that has also been observed following extended opioid use and withdrawal. The hypersensitivity of the extended amygdala may motivate persons suffering from chronic non-cancer pain or opioid withdrawal to actively seek and take opioids to alleviate their negative emotions and psychological perceptions of pain. Emerging therapeutic strategies to mitigate stress and opioid misuse within the chronic pain populations are discussed.

This article is complemented by the review by Massaly et al., which tackles the issue of chronic pain and opioid misuse from a preclinical and mechanistic viewpoint. Like the previous review article, the authors describe the shared mechanisms between chronic pain and stress, but with a focus on the mesolimbic dopamine system. Driven by top-down control from the kappa opioid receptor system, the authors argue that alterations in dopaminergic signaling in chronic pain and stress generate long-term modifications in the reinforcing properties of opioids leading to increased abuse liability.

Pain is a complex experience that engages cognitive, emotional, and sensory processing. Blocking sensory nociceptive transmission circuits and engaging descending modulatory circuits originating in midbrain and brainstem are primary mechanisms responsible for opioid-induced pain relief. Indeed, one could argue that the sensory component of acute pain is the primary driver of the pain experience because nerve blocks and topical anesthetics provide significant pain relief. But this doesn’t apply to chronic pain. The reviews and original papers in this research topic provide strong evidence that the cognitive and emotional components of pain are the underpinnings of the chronic pain experience. Indeed, this difference likely accounts for the lack of new analgesic drugs that have made it to market and the failed clinical trials of promising new analgesics for treating chronic pain. It also argues that preclinical and clinical pain research laboratories, as well as pharmaceutical and biotech companies, must move beyond sensory-evoked measures of nociception as predictors of novel analgesics for alleviating chronic pain. Focusing on mechanisms within limbic circuitry will deepen our understanding of the complex condition of chronic pain and invigorate new approaches for alleviating chronic pain.

## Author Contributions

AT and CC conceived the idea and wrote the manuscript.

## Conflict of Interest Statement

The authors declare that the research was conducted in the absence of any commercial or financial relationships that could be construed as a potential conflict of interest.

